# The Relation of Alcoholism to Suicide

**Published:** 1902-03-15

**Authors:** 


### THE RELATION OF ALCOHOLISM TO SUICIDE.

An interesting contribution on the above subject from the pen of Dr. W. C. Sullivan, of Her Majesty's Prison at Pentonville, has appeared in a recent number of the *Journal of Mental Science* (April, 1900). An attempt is made "to determine the rôle which alcoholism may have played in the late increase of suicide in England, and at establishing the distinctive characters which constitute the type of alcoholic suicide." In the classical work of Dr. Magnus Huss, that distinguished observer states "that the suicidal impulse is a more frequent accompaniment of the melancholia of drunkards than of melancholia from other causes; and further, that among the uneducated classes suicide frequently follows on the disordered emotional tone, which, sooner or later, results from the abuse of alcoholic liquors." The majority of observers have adopted the view which regards alcoholism as one among the more potent causes of suicide. Thus Lunier, whose views are in the main adopted by Morselli, found in the different departments of France that a high average consumption of alcohol was invariably accompanied by a high rate of suicide. On the other hand Baer, whose authority on the question is undoubted, has demonstrated the absence of such a correspondence in Prussia, and cites also as a counter-argument the case of Sweden, where decrease in alcoholism has failed to arrest the upward movement of suicide. Grotjahn, who regards alcoholism mainly as a direct result of the condition of the proletariat under the capitalistic *régime*, while admitting a certain coincidence in the regional and periodic distribution of alcoholism and suicide, regards their relationship as that of co-effects of a common cause. Dr. Sullivan, after a brief consideration of these and other writers, concludes that alcoholism is but one of the several causes of suicide, and that its relative importance as a suicidigenous factor differs in different places and at different epochs. Taking as the measure of alcoholism the deaths attributed to intemperance, which amounted to 35 per million in the Registrar-General's returns for 1867, and which has steadily risen in successive periods till it stood at 76 per million in the returns for 1897, it appears that an increase of alcoholism in the 30 years (1867-1897) amounts to over 100 per cent. "The records of coroners' verdicts of 'death from excessive drinking' shows a similar increase." A careful study also of the returns in the appendix to Mr. Whittaker's Memorandum, published with the report of the Licensing Commission, gives the average consumption per head of beer and spirits in the United Kingdom for the years 1842-98. From the figures given it appears that such consumption "has of late years shown an upward tendency, so marked as to bring the figures for recent years almost up to the level reached in the early seventies, when (coincident with the feverish industrialism of the period, and the enormous multiplication of licenses) English drinking habits attained their highest point." As regards the recent movement of suicide the Registrar-General's returns show that in England there has been a steady increase in the suicide rate during the last three decennial periods. Thus the proportion per million, which in the decennium 1861-70 stood at 65, rose in the following decade to 70; and in the decade 1881-90 amounted to 77, representing an increase of over 18 per cent. on the figures of the first-named period. As regards attempts to commit suicide, there has been a similar and even more decided increase. Thus in the period 1867-71 the number of cases of attempted suicide amounted to 35·5 per million inhabitants; in each successive quinquennial period it stood higher, and in the period 1892-96 it rose to 57·9 per million—an increase of over 78 per cent. on the first-cited figures. Diagrams are given from the yearly returns, 1874-97, to illustrate the increase of both actual suicides and suicidal attempts, and Dr. Sullivan shows "firstly, that suicidal tendencies have grown in a degree entirely out of proportion to the increase of population; and secondly, that their growth has been much more considerable in the category of suicidal attempts than in that of actual suicides." As regards age and sex it is found that the proportion of suicides by persons aged over 45 years is 55·6 per cent., the period of maximum incidence being the decade 45-55 years of age. On the other hand the proportion of suicidal attempts among adult males fined by law for such showed that the period of maximum incidence was the decade 30-40. A similar contrast is found as regards females; the maximum incidence in actual suicides being found in the decade 35-45, while attempts to commit suicide prevail in the period 21-30. Hence it appears that abortive suicidal attempts tend to operate at a relatively early age. From Dr. Tatham's tables of occupational groups with a high rate of alcoholism, viz., publicans, butchers, coach and cab service, commercial travellers, hairdressers, and musicians, it appears that these groups also furnish a relatively high rate of suicide. From a careful discussion of the facts Dr. Sullivan draws the following conclusions:—*Firstly*, that the recent increase of suicide has coincided with a considerable development of abortive suicidal attempts. These attempts, in such of their characters as are ascertainable, have approximated to the type of alcoholic suicide, thereby confirming the clinical evidence which attributes to alcoholism the chief rôle in the genesis of abortive suicidal impulses. *Second*, that this increase of suicide has been in a large degree related to the influence of alcoholism, an influence which in the same period (as mortality statistics attest) has tended to augment. *Third*, that there is a type of alcoholic suicide, with characteristics distinguishing it from suicide of other causation, and that, as compared with cases of deliberate and coordinated suicide due to other causes, alcoholic suicide is found to be more impulsive, more directly and immediately related to organic conditions in the individual. *Fourth*, the chronic intoxication by alcohol, as we observe it clinically, produces generalised disorders of visceral function throughout the economy, whence there results an alteration and disturbance of those organic stimuli which form the groundwork of our personality; those stimuli whose activity, as Maudsley puts it, is "even of more consequence in determining the tone of our feeling or of our disposition and the character of our impulses than that activity which follows impressions received from the external world." The depressed emotional tone thereby induced prepares the suicidal impulse, which issues in action when a supervening increase of intoxication has still further lowered the level of function in the brain.

